# Consulting Surgeon to the Cincinnati Eye Infirmary

**Published:** 1828-10

**Authors:** 


### III. Original.

21. *Cincinnati Eye Infirmary.* At the request of the Surgeon of the Infirmary, the Visitors have appointed Professor Cobb, a consulting Surgeon of this public charity.

